# Test–retest reliability of the Italian version of the M‐BACK questionnaire to assess the barriers, attitudes, confidence, and knowledge of mental health staff regarding metabolic health of psychiatric patients

**DOI:** 10.1002/brb3.1491

**Published:** 2019-12-25

**Authors:** Attilio Carraro, Erica Gobbi, Marco Solmi, Andrew Watkins, Philip B. Ward, Simon Rosenbaum

**Affiliations:** ^1^ Faculty of Education Free University of Bozen Bozen Italy; ^2^ Casa di Cura Parco dei Tigli Villa di Teolo Padova Italy; ^3^ Department of Biomedical Sciences University of Padua Padua Italy; ^4^ Department of Neurosciences University of Padova Padova Italy; ^5^ Keeping the Body in Mind Program Bondi Centre South Eastern Sydney Local Health District Sydney NSW Australia; ^6^ Faculty of Health University of Technology Sydney NSW Australia; ^7^ School of Psychiatry University of New South Wales Sydney NSW Australia; ^8^ Schizophrenia Research Unit Ingham Institute for Applied Medical Research South Western Sydney Local Health District Liverpool Hospital Sydney NSW Australia; ^9^ Black Dog Institute Prince of Wales Hospital Sydney NSW Australia

**Keywords:** evaluation tool, metabolic syndrome, physical health, reliability, severe mental illness

## Abstract

**Objectives:**

The Metabolic‐Barriers, Attitudes, Confidence, and Knowledge Questionnaire (M‐BACK) was developed to determine the barriers, attitudes, confidence, and knowledge of mental health practitioners regarding the metabolic health of patients in order to determine the efficacy of targeted training interventions. This study aimed to validate the Italian version of M‐BACK questionnaire (M‐BACK‐IT) and to determine the test–retest reliability.

**Methods:**

The M‐BACK questionnaire was translated into Italian and back‐translated using an established protocol. In order to determine the test–retest reliability of the instrument, mental health professionals were recruited from a private psychiatric hospital located in northeast Italy and completed the questionnaire on two separate occasions, seven days apart. Intraclass correlation coefficients (ICC) were calculated for the total score, as well as each of the four M‐BACK domains.

**Results:**

Thirty mental health professionals (4 psychiatrists, 9 psychologists, 12 nurses, and 5 exercise specialists) completed the M‐BACK‐IT. ICCs ranged from 0.58 to 0.94.

**Conclusions:**

The test–retest reliability of the M‐BACK‐IT demonstrated comparable results to the English version. The M‐BACK‐IT is a reliable measure to assess key elements of practitioners' perceptions of the barriers, their knowledge, attitudes, and confidence regarding metabolic monitoring and intervention in people with mental illness.

## Graphical abstract

This study aimed to validate the Italian version of M‐BACK questionnaire (M‐BACK‐IT) and to determine the test–retest reliability. Thirty mental health professionals (4 psychiatrists, 9 psychologists, 12 nurses, and 5 exercise specialists) completed the M‐BACK‐IT, intraclass correlation coefficients (ICC) were calculated for the total score, and each of the four M‐BACK domains, ICCs, ranged from 0.58 to 0.94.

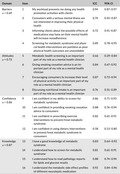

## 1 INTRODUCTION

People living with mental illness experience an increased mortality rate of approximately 2.2 times that of the general population (Walker, McGee, & Druss, 2015). Those living with severe mental illness (SMI), such as schizophrenia, bipolar disorder, and major depressive disorder, have a reduced life expectancy of approximately 15 years (Hjorthøj, Stürup, McGrath, & Nordentoft, 2017), primarily due to preventable and premature cardiometabolic diseases (Correll et al., 2017). People with SMI have a 53% higher risk of having cardiovascular disease (CVD), are 78% more likely to develop CVD in the future, and are roughly 85% more likely to die from CVD in comparison with the general population (Correll et al., 2017). Further, people with SMI are more likely than the general population to meet criteria for metabolic syndrome (Vancampfort et al., 2015), a cluster of risk factors including abdominal obesity, dyslipidemia, hypertension, and insulin resistance (Zimmet et al., 2007), which is a significant contributing factor to the overall burden of CVD in this population. For example, among people with psychosis the incidence of metabolic syndrome has been documented as high as 61% which is approximately 2–3 times higher than the general population (Morgan et al., 2013).

Although there is only limited available data from Italian samples (Carrà et al., 2014; Clerici et al., 2014), internationally, the issues of physical health inequalities for people living with mental illness and the subsequent premature mortality have been identified as a public health crises (Thornicroft, 2011), with increasing calls for urgent action (Stewart, 2015; Suetani, Whiteford, & McGrath, 2015). A 2017 Forum on the need for a comprehensive approach to addressing the excess mortality in people with SMI in collaboration with the World Health Organization identified a multilevel intervention framework and the priorities for clinical practice, policy, and research agendas, including a focus across both individual factors (e.g., modifiable behavioral risk factors such as smoking and low levels of physical activity), health systems (including service delivery and human resources) and the social determinants of health (Liu et al., 2017). Additionally, in Australia and the United Kingdom a number of high‐level policy‐based documents have been developed including guidance from the Royal Australian and New Zealand College of Psychiatrists (The Royal Australian & New Zealand College of Psychiatrists, 2015) and a briefing report from Public Health England (Public Health England, 2018).

Mental health staff are well positioned to play a key role in providing metabolic health care (Happell et al., 2011). However, while mental health nurses acknowledge that they have a role in addressing physical health needs (Robson, Haddad, Gray, & Gournay, 2012), they may not have the confidence or skills to identify and manage metabolic comorbidity experienced by people with mental illness, which has led to recommendations for specific training in this area (Howard & Gamble, 2011; Nash, 2005; Terry & Cutter, 2013), with evidence increasing that brief training can lead to improvements in the rate of metabolic monitoring within inpatient settings (Rosenbaum et al., 2014). In order to help determine the effectiveness of such training initiatives and to inform the design and targeting of such training, the Metabolic‐Barriers, Attitudes, Confidence, and Knowledge Questionnaire (M‐BACK) was developed (Watkins et al., 2017). The M‐BACK is a 16‐item instrument addressing the knowledge, confidence, attitudes, and practice barriers of mental health staff in relation to metabolic health (Watkins et al., 2017). Responses are rated on a five‐point Likert‐type scale ranging from Strongly Disagree (scoring 1) to Strongly Agree (scoring 5). Each domain is composed of four items, with a minimum score of 4 and a maximum score of 20.

Domain 1: Barriers (Questions 1–4). Items address barriers to metabolic screening and intervention, including, workload, consumer interest, conflict with mental health goals, and inability to effect change. These questions are negatively posed, with the scoring reversed.
Domain 2: Attitudes (Questions 5–8). Items investigate attitudes, including toward metabolic monitoring, the provision of smoking cessation advice, physical activity, and nutritional intake.
Domain 3: Confidence (Questions 9–12). Items assess the confidence of respondents in providing interventions to prevent or treat metabolic health including smoking cessation, physical activity, and nutritional interventions.
Domain 4: Knowledge (Questions 13–16). Items assess knowledge of metabolic health, screening, interpreting pathology reports, and understanding of the metabolic side effects of neuroleptic medication.

The aims of this study were to determine the psychometric properties of the M‐BACK into Italian (M‐BACK‐IT) using established translation protocol and to determine the test–retest reliability of the instrument.

## 2 METHOD

The development of the English version of the M‐BACK has been reported elsewhere and briefly involved a comprehensive literature review, and consultation and review by a multi‐disciplinary panel of experts including clinicians, educators, and researchers (Watkins et al., 2017). Pilot testing was subsequently conducted with mental health nurses and structured feedback on the content, and usability of the tool was obtained. Feedback was collated, and consensus on the final version was then achieved through group discussion with the expert panel.

The translation process followed established protocol and involved a series of steps including (a) forward translation, (b) reconciliation, (c) back translation and review, (d) harmonization, and (e) cognitive debriefing and finalization.

The M‐BACK questionnaire is intended to assess change over time and to detect change in the items following specific education or training targeting mental health professionals (Watkins et al., 2017). Internal consistency with Cronbach's alpha was tested and, as per the original validation study, test–retest for each item was determined by calculating intraclass correlation coefficients using a two‐factor mixed effects model (Shrout & Fleiss, 1979). Likert‐type items are typically considered as a continuous variable, and intraclass correlation coefficient (ICC) is the recommended method of assessing test–retest data for continuous data (Rankin & Stokes, 1998). Using ICC can determine the proportion of total variance that occurs between time points (Shrout & Fleiss, 1979).

Ninety‐two mental health professionals from a private psychiatric hospital in northeast Italy were invited to complete the questionnaire. There were no other exclusion criteria. Participants were informed of the aims of the study that participation was completely voluntary, and there were no consequences for not participating. Participants completed a signed consent form. Thirty‐eight professionals (41.3% of the invited staff) completed the questionnaire on the first occasion. Eight participants were unable to complete the questionnaire at the second time point, due to time constraints and clinical responsibilities. The majority of the sample who completed the questionnaire on the two occasions (seven days apart) were female (70%) and aged <50 years (80%).

Data were reviewed to ensure completeness. Cronbach's alpha and ICCs were calculated for the total score, as well as each of the four domains (Barriers, Attitudes, Confidence, and Knowledge). ICCs were interpreted based on Landis and Koch (1977), with ICCs of below 0.4 (poor to fair), 0.41–0.6 (moderate), 0.61–0.80 (excellent), and 0.81–1 (almost perfect) (Landis & Koch, 1977). The questionnaire was analyzed for individual items in addition to the four domain areas.

The study was approved by the University of Padua, Department of Biomedical Sciences Human Research Ethics Committee (HEC‐DSB/01‐19). Anonymity was ensured by using appropriate codes and storing demographic data separate from the completed questionnaires.

## 3 RESULTS

Thirty staff from a private psychiatric hospital in Northern Italy participated in this study. Participants had different background and roles, 40% were nurses, 30% psychologists, 17% exercise specialists, and 13% psychiatrists. In total, 70% of participants (*n* = 21) were female and the majority (80%) were aged <50 years. They completed the M‐BACK‐IT on two occasions, seven days apart.

The internal consistency of the questionnaire resulted in acceptable to good values with alphas ranging from 0.69 to 0.87 for the different domains (see Table 1 for the detailed values) and *α* = 0.71 for the total score.

**Table 1:** Cronbach's alpha and Intraclass correlation coefficients for M‐BACK‐IT domains

Domain	Item		ICC	95% CI
Barriers *α* = 0.69	1	My workload prevents me doing any health promotion activities with clients	0.94	0.87–0.97
	2	Consumers with a serious mental illness are not interested in improving their physical health	0.74	0.45–0.87
	3	Informing clients about the possible effects of medications may have on their mental health will increase nonadherence	0.72	0.41–0.87
	4	Screening for metabolic syndrome and physical health interventions are pointless as poor physical health outcomes are unavoidable	0.89	0.78–0.95
Attitudes *α* = 0.73	5	Metabolic health screening is an important part of my role as a mental health clinician	0.66	0.29–0.84
	6	Giving smoking cessation advice is an important part of my role as a mental health clinician	0.84	0.67–0.92
	7	Encouraging consumers to increase their level of physical activity is an important part of my role as a mental health clinician	0.87	0.72–0.94
	8	Discussing nutritional intake is an important part of my role as a mental health clinician	0.76	0.51–0.89
Confidence *α* = 0.86	9	I am confident in my ability to screen for metabolic syndrome	0.86	0.71–0.93
	10	I am confident in providing smoking cessation advice to consumers	0.88	0.76–0.94
	11	I am confident in prescribing exercise interventions to prevent/treat metabolic syndrome	0.82	0.61–0.91
	12	I am confident in using dietary interventions to prevent/treat metabolic syndrome in consumers	0.58	0.13–0.80
Knowledge *α* = 0.87	13	I have a good knowledge of metabolic syndrome	0.83	0.64–0.92
	14	I understand how to screen for metabolic syndrome	0.81	0.61–0.91
	15	I understand how to read pathology reports for lipids and glucose results	0.88	0.74–0.94
	16	I understand the metabolic side effect profiles of different neuroleptic medication	0.92	0.84–0.96

ICC correlations for individual items ranged from 0.58 to 0.94 (see table 1).

### 3.1 Total score

A high degree of reliability was found between the total M‐BACK‐IT scores at both time points. The single measure ICC was considered excellent, 0.87 (95% CI 0.72–0.94). The mean between day variation was 0.77 ± 4.32.

### 3.2 Barriers

The ICCs for the barrier items ranged from 0.72 (Item 3 “Informing clients about the possible effects of medications may have on their mental health will increase nonadherence”) to 0.94 (Item 1 “My workload prevents me doing any health promotion activities with clients”). The ICC for this domain of the M‐BACK‐IT was 0.89 (95% CI 0.77–0.95).

### 3.3 Attitudes

The ICCs for the attitude items ranged from 0.66 (Item 5 “Metabolic health screening is an important part of my role as a mental health clinician”) to 0.87 (Item 7 “Encouraging consumers to increase their level of physical activity is an important part of my role as a mental health clinician”). The ICC of the Attitude domain was 0.86 (95% CI 0.66–0.92).

### 3.4 Confidence

The ICCs for the confidence items ranged from 0.58 (Item 12 “I am confident in using dietary interventions to prevent/treat metabolic syndrome in consumers”) to 0.88 (Item 10 “I am confident in providing smoking cessation advice to consumers”). The domain ICC was 0.88 (95% CI 0.75–0.94).

### 3.5 Knowledge

The ICCs for the knowledge items ranged from 0.81 (Item 14 “I understand how to screen for metabolic syndrome”) to 0.92 (Item 16 “I understand the metabolic side effect profiles of different neuroleptic medication”). The Knowledge domain ICC was 0.91 (95% CI 0.81–0.96).

## 4 DISCUSSION AND CONCLUSION

This paper describes the translation, and test–retest reliability of the M‐BACK‐IT, a novel tool designed to assess the attitudes, confidence, and knowledge of mental health practitioners in providing metabolic care to people experiencing mental illness and the perceived barriers in the delivery of appropriate interventions.

The M‐BACK plays an important role in the bridging the established implementation gap between research and practice, for example by gaining an understanding of the barriers to the provision of metabolic screening and interventions, results of the M‐BACK can inform the design and delivery of subsequent training and education. It is also an important tool by which the effectiveness of training and education for mental health professionals and other initiatives of mental health services to address the important area of metabolic health care can be evaluated. For example, using pre–post design, it also enables evaluation of the effectiveness of education regarding metabolic health care through pre‐ and post‐training testing. Similarly, the M‐BACK can be used to determine the effectiveness of staff‐based interventions for mental health professionals (Fibbins, Ward, Watkins, Curtis, & Rosenbaum, 2018).

The Italian version of the questionnaire demonstrated acceptable to good internal consistency and good test–retest reliability, with an ICC of 0.87 for the total M‐BACK‐IT score and 0.86–0.91 for each of the four domains (Barriers, Attitudes, Confidence, and Knowledge).

Test–retest reliability analysis will rarely achieve perfect results (Wikman & Wärneryd, 1990). Within the domains, the greatest variance was found within the Attitude and Confidence domains and least variance within the knowledge domain. Item 12 in the Attitude domain “I am confident in using dietary interventions to prevent/treat metabolic syndrome in consumers” had the lowest ICC of any item throughout the questionnaire (0.58), although this was still classified as moderate according to the criteria of Landis and Koch (1977) (Landis & Koch, 1977). Possible explanations for why some items in a questionnaire may have greater variability using test–retest reliability can be due to a change of opinion or research undertaken by a participant in between test dates, perhaps even prompted from completing the questionnaire (Wikman & Wärneryd, 1990).

Given the acceptable outcomes of all results in the test–retest, it was determined that no changes to questions were required to ensure instrument validation. The current paper contributes to the recommendations of the recent Lancet Psychiatry Commission: Ensuring culturally appropriate scales are available in languages other than English is of key significance, given increasing international calls for a global approach to addressing the poor physical health of people with mental illness. Culture change and upskilling mental health clinicians regarding lifestyle interventions are critical, and therefore, having language‐specific, validated tools to measure change and progress is imperative (Firth et al., 2019).

The M‐BACK‐IT questionnaire is not without limitations, and further research with a larger and more diverse sample of mental health professionals working across different settings is recommended. Determining whether responses vary as a consequence of professional background (i.e., investigating differences between psychiatrists, psychologists, and nursing staff) and specific characteristics of different mental health services (e.g., those treating inpatients or outpatients) would be of clinical relevance. This may also inform whether discipline‐specific or setting‐oriented versions of the instrument are required.

The M‐BACK‐IT tool seems to be a valid and reliable instrument to measure the attitudes, confidence, and knowledge of mental health staff in metabolic health and their perceptions of barriers to delivering such metabolic health care. There is a clearly identified need for initial and in‐service training and education for mental health staff in regard to the metabolic health of mental health consumers.

## CONFLICT OF INTEREST

The authors declare that they have no competing interests.

## Data Availability

The data that support the findings of this study are available from the corresponding author upon reasonable request.
